# Quantifying and Forecasting Emission Reductions in Urban Mobility: An IoT-Driven Bike-Sharing Analysis

**DOI:** 10.3390/s25072163

**Published:** 2025-03-28

**Authors:** Manuel Uche-Soria, Bernardo Tabuenca, Gonzalo Halcón-Gibert, Yilsy Núñez-Guerrero

**Affiliations:** 1 Department of Engineering Organization, Business Administration and Statistics, Universidad Politécnica de Madrid, 28006 Madrid, Spain; m.uche@upm.es (M.U.-S.);; 2 Department of Computer Systems, Universidad Politécnica de Madrid, 28031 Madrid, Spain

**Keywords:** bike-sharing systems, gas emissions, IoT sensors, air quality forecasting, machine learning, urban mobility, environmental impact

## Abstract

The growing urgency to address urban air quality and climate change has intensified the need for sustainable mobility solutions that mitigate vehicular emissions. Bike-sharing systems (BSSs) represent a viable alternative; however, their precise environmental impact remains insufficiently explored. This study quantifies and forecasts reductions in CO_2_ and NO_x_ emissions resulting from BSS usage in Madrid by integrating real-time IoT sensor data with an advanced predictive model. The proposed framework effectively captures nonlinear and seasonal mobility and emission patterns, achieving high predictive accuracy while demonstrating significant energy savings. These findings confirm the environmental benefits of BSSs and provide urban planners and policymakers with a robust tool to extend and replicate this analysis in other cities, fostering sustainable urban mobility and improved air quality.

## 1. Introduction

Urban mobility significantly influences the environmental impact of transportation, especially in densely populated cities where traffic congestion and greenhouse gas (GHG) emissions exacerbate climate change and air pollution. Growing concerns about urban air quality have led to the search for efficient and eco-friendly transportation solutions [1,2,3,4]. Among these, bike-sharing systems (BSSs) have proven to be an effective strategy for reducing private vehicle dependency and promoting low-carbon mobility [1].

Despite the benefits of BSSs, challenges such as inefficient station distribution, fluctuating demand, and limited integration with other transport modes remain critical. Recent advances in IoT-enabled environmental monitoring have demonstrated the potential of wireless sensor networks to provide real-time data for urban air quality assessment, optimize fleet distribution, and improve operational efficiency [5,6,7,8]. In particular, Puyol and Baeza [8] demonstrated how IoT networks can enhance the real-time monitoring and optimization of bike-sharing systems, ensuring more efficient usage, reduced operational costs, and improved safety on journeys by rewarding good user behavior with a reduction in rental cost. Their work highlights the importance of integrating sensor data with predictive models, which aligns closely with this study’s approach to quantifying emission reductions through BSSs. Furthermore, the integration of machine learning models has facilitated the prediction of mobility patterns and environmental impact, allowing for data-driven decision-making in urban planning [9,10]. Beyond operational enhancements, AI and IoT technology are also being leveraged for security improvements. Karthika et al. [11] introduced a security framework that combines IoT tracking with Support Vector Machine (SVM) models to detect and prevent bicycle theft, thereby increasing system reliability. This approach demonstrates how IoT applications in BSSs extend beyond mobility optimization to ensure secure and sustainable operations.

Sustainability in urban environments and its relationship with the use of bike-sharing services is a topic of great interest, not only for our daily environment, as a key element of urban sustainability policies, but also in the scientific literature. Various environmental, economic, and social factors shape this relationship. BSSs generate large datasets that can be analyzed to understand urban mobility patterns, supporting infrastructure planning and policy-making [9]. Guo et al. [12] examined how the integration of bicycle docking stations with public transport can influence passenger attitudes and facilitate greater use of public transport, fostering a more sustainable transport system. Similarly, Guo and He [13] analyzed how the built environment affects the integration of dockless bike-sharing with subway systems, highlighting the importance of urban design in promoting bicycle use in Shenzhen (China). Another key study by Zhang et al. [14] proposed a model to optimize the distribution of shared bicycles during morning peak hours, improving traffic efficiency and reducing carbon emissions. Research by Wang et al. [15] explored the influence of built environment factors on bike-sharing usage through geographic detector models, using 6.5 million bike-share orders in Shanghai (China). Their findings provided urban designers with strategies to enhance bicycle use by integrating key environmental variables.

Beyond mobility efficiency, studies have quantified the broader impacts of bike-sharing programs on the economy, energy consumption, environmental protection, and public health [16]. These programs contribute significantly to carbon neutrality by reducing dependence on fossil fuels [17]. Furthermore, Tao and zuo Zhou [18] identified several sustainability benefits of dockless bike-sharing services, such as improved resource utilization and reduced GHG emissions. In a study of Shanghai’s bike-sharing program, Zhang and Mi [19] estimated that in 2016, the system saved 8358 tons of gasoline and reduced CO_2_ emissions by 25,240 tons and NO_x_ emissions by 64 tons. Similarly, Chen et al. [20] conducted a retrospective analysis of bike-sharing in New York (USA), estimating energy savings and emission reductions from historical data (2014–2017), albeit without predictive modeling. Using service usage data from Lisbon (Portugal), Raposo and Silva [21] estimated that the e-bike sharing system could prevent 36 tons of GHG emissions annually and reduce energy consumption by 451 GJ. Beyond emissions reduction, Sun and Ertz [22] analyzed how free-flowing bike-sharing (FFBS) optimized resource use compared to station-based bike-sharing (SBBS) and privately owned bicycles, revealing significant reductions in aluminum, steel, plastic, and rubber consumption. Finally, Lu et al. [23] conducted simulations showing that integrating free bikesharing with transit systems could save USD 1.5 million per year in transportation damage costs and prevent 22 premature deaths annually by shifting mobility towards cycling and walking in Taipei (Taiwan).

Those studies highlight the multifaceted benefits of bike-sharing: (i) improving resource utilization, (ii) reducing greenhouse gas emissions, (iii) decreasing energy consumption, and (iv) enhancing public health. Unlike previous studies that have assessed the environmental impact of BSSs using historical data or indirect estimations, this work integrates real-time IoT sensor data with advanced predictive models to capture nonlinear mobility patterns and their effects on pollution. Additionally, the proposed approach enables the replicability of the model in other cities, providing an adaptable framework for decision-making in sustainable urban planning. The study presented in this article quantified the environmental impact of the bike-sharing system in Madrid (Spain) by leveraging IoT sensors and predictive machine learning models to quantify reductions in CO_2_ and NO_*x*_ emissions. Through mobility data analysis, this research sought to assess the influence of BSS infrastructure on air quality and explore measures to enhance its environmental benefits. Accordingly, this study formulated the following research questions (RQ):

- RQ1: What is the state-of-the-art of machine learning models for estimating GHG emissions in large cities?
- RQ2: How can IoT sensors and predictive machine learning models be used to accurately quantify the environmental impact of the bike-sharing system in Madrid, specifically in terms of CO_2_ and NO_*x*_ emission reductions?

This research is structured as follows: Section 2 addresses RQ1, reviewing the literature related to the use of IoT in urban mobility and the modeling of GHG emissions. Section 3 and Section 4 address RQ2, analyzing mobility patterns to explore the potential of BSS data to anticipate GHG emissions in large cities. Finally, Section 5 discusses the findings and provides insights for further research.

## 2. Machine Learning Models for Urban GHG Prediction

The growing concern about greenhouse gas emissions in urban areas has led to the development of advanced modeling techniques for their prediction and mitigation. Among sustainable mobility solutions, bicycle-sharing services have gained prominence due to their potential to reduce transportation-related emissions. However, accurately estimating their real impact remains a challenge, necessitating robust predictive models. This chapter explores the state of the art in the predictive modeling of GHG emissions in urban settings, with a specific focus on integrating BSSs into these models.

Following the first research question (RQ1), this review follows three main research paths: (i) the application of artificial intelligence in urban pollution prediction, (ii) the use of neural networks to model air pollution in cities with BSSs, and (iii) AI-driven applications specifically designed for BSSs. To identify relevant studies, a literature review was conducted using the terms “predictive model”, “bicycle sharing service”, and “artificial intelligence” across major academic databases, covering research published between 1995 and 2024. While extensive research has applied big data to quantify urban pollutants, no studies were found that explicitly leveraged predictive models to estimate GHG emissions in large cities through BSSs.

**Figure 1:**
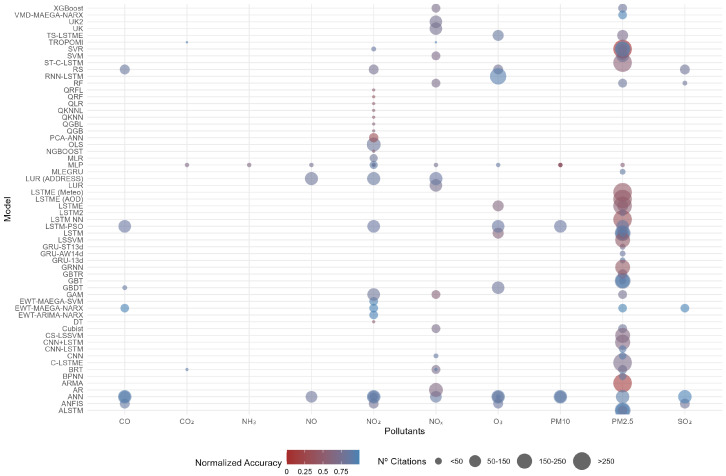
ML models assessing urban pollutants.

Figure 1 provides an overview of the classification of existing models based on four key dimensions: the machine learning model used (Y-axis), the accuracy of the model (color gradient from 0/red to 1/blue), the pollutants observed (X-axis), and the number of citations in Google Scholar. Additional details, including specific references and model performance metrics, are presented in Table A1 in Appendix A. Analysis of Figure 1 shows that modeling efforts are predominantly focused on ${\mathrm{PM}}_{2.5}$, which emerged as the most studied pollutant, reflecting a strong research interest and a high number of citations for related studies. Other pollutants such as ${\mathrm{NO}}_{2}$, ${\mathrm{NO}}_{x}$, and $O_{3}$ have also been the subject of research, though to a lesser extent. In terms of model performance, techniques like *XGBoost*, *VMD-MAEGA-NARX*, *UK2*, and *TS-LSTM* demonstrate outstanding accuracy, particularly in predicting ${\mathrm{NO}}_{x}$ and ${\mathrm{PM}}_{2.5}$, achieving $R^{2}$ values close to 0.75. This suggests that advanced machine learning methods, including neural networks and hybrid models, are well suited for handling these pollutants in urban settings. The number of citations associated with each study further indicates that while many high-accuracy models receive substantial academic recognition—especially those focusing on ${\mathrm{PM}}_{2.5}$ and ${\mathrm{NO}}_{x}$—some high-performing models remain undercited, potentially pointing to gaps in dissemination or emerging areas for further exploration. Interestingly, only one of the models incorporates satellite-based prediction, underscoring a prevailing trend in the literature favoring machine learning-driven approaches over remote sensing techniques for urban pollution modeling.

### Comparative Analysis of Predictive Models for Urban Air Pollution

Models based on *satellite observations* [24] have proven to be a reliable approach for estimating ${\mathrm{NO}}_{x}$ and ${\mathrm{CO}}_{2}$ emissions in large urban areas. In a recent study, researchers successfully reduced the uncertainty of daily ${\mathrm{NO}}_{x}$ and ${\mathrm{CO}}_{2}$ emission estimates in Wuhan (China) to 31% and 43%, respectively. Furthermore, these estimates were validated against bottom-up emission inventories, revealing deviations of less than 3% on average for the model year. Magazzino et al. [25] highlighted the potential of machine learning models in analyzing the relationship between renewable energy production and ${\mathrm{CO}}_{2}$ emissions, emphasizing AI’s capacity to model complex environmental systems. This approach also facilitates the quantification of environmental benefits associated with sustainable transportation solutions. Among machine learning techniques, algorithms such as *Random Forest (RF)* and *Boosted Regression Trees (BRTs)* have demonstrated high efficacy in predicting hourly PM2.5 and ${\mathrm{NO}}_{x}$ concentrations in Hong Kong (China), with *RF* exhibiting the best performance [26]. Additionally, the application of *Gradient Boosting* regression models has enabled highly accurate predictions of ${\mathrm{NO}}_{x}$ and ${\mathrm{CO}}_{2}$ emissions from diesel vehicles under different driving scenarios [27]. *ANFIS (Adaptive Neuro-Fuzzy Inference System)* and *semi-experimental nonlinear regression* models have been applied in air pollution prediction. In this context, Zeinalnezhad et al. [28] demonstrated that the *ANFIS* model exhibited superior accuracy compared to the *semi-experimental regression* model across all pollutants, achieving higher $R^{2}$ values and indicating a better alignment between the predicted and observed data.

*Artificial Neural Networks (ANNs)*, when combined with uncertainty analysis using Monte Carlo simulations, have also demonstrated a strong correlation in predicting pollutants such as ${\mathrm{NO}}_{x}$, ${\mathrm{NO}}_{2}$, and CO in urban environments [29]. In Münster (Germany), *ANNs* have been successfully implemented to forecast hourly concentrations of multiple pollutants, particularly achieving reliable results for NO, ${\mathrm{NO}}_{2}$, and ${\mathrm{NO}}_{x}$ [30].

*Ordinary regression* and *time series autocorrelation* models have also been employed to estimate the hourly concentrations of ${\mathrm{NO}}_{x}$ and ${\mathrm{NO}}_{2}$, emphasizing the role of meteorological factors such as wind speed and chemical reactions in London (UK) [31]. However, these models have yielded suboptimal results, particularly in the case of the *autoregressive* model, which reported an $R^{2}$ value of only 0.65.

Regarding *quantile regression methods*, Vasseur and Aznarte [32] conducted a study in Madrid (Spain), concluding that *quantile gradient boosted trees* were the most effective model for predicting ${\mathrm{NO}}_{2}$ pollution levels. Nevertheless, they also found that simpler approaches, such as *quantile nearest neighbors* combined with *linear regression*, provided comparable results while reducing computational complexity and training time.

Liu et al. [33] introduced a hybrid approach combining three algorithms—*Empirical Wavelet Transform (EWT)*, *Modified Adaptive Genetic Algorithm (MAEGA)*, and *Nonlinear Autoregressive with Exogenous Inputs (NARX)*—resulting in the *EWT-MAEGA-NARX* model, which demonstrated effective performance in predicting air pollutant concentrations in Beijing (China). Similarly, Mao et al. [34] proposed the *TS-LSTME (Temporal Sliding Long Short-Term Memory Extended)* model for air quality prediction in the Jing-Jin-Ji region of (China), focusing on ${\mathrm{PM}}_{2.5}$ and $O_{3}$ concentrations. The results indicated that *TS-LSTME* achieved high predictive accuracy, with an $R^{2}$ coefficient of 0.72 for ${\mathrm{PM}}_{2.5}$ and 0.86 for $O_{3}$, outperforming other models such as *LSTME* and *LSTM*. This deep learning approach, which integrates meteorological and temporal data, has proven to be effective for long-term forecasting and can be extended to the prediction of multiple air pollutants.

Predictive models such as *linear regression* and *random forest* have been employed to estimate spatial variations in air pollution within urban environments. However, improvements in evaluation methods and interpretability remain necessary [35]. Among the recent advancements, the proposed *C-LSTME* model has demonstrated superior performance compared to state-of-the-art models for air pollution forecasting across different temporal scales and regional contexts [36]. Similarly, Chang et al. [37] introduced a hybrid machine learning approach aimed at enhancing the predictive accuracy of ${\mathrm{PM}}_{2.5}$ and ${\mathrm{PM}}_{10}$ concentrations. Their study evaluated multiple models, including *Gradient-Boosted Tree Regression (GBT)*, *Support Vector Regression (SVR)*, and *Long Short-Term Memory (LSTM)*, along with an optimized variant, *LSTM2*. The results indicated that *GBT* exhibited the highest accuracy, achieving an $R^{2}$ of 0.83 for ${\mathrm{PM}}_{2.5}$ predictions, followed by *SVR* and *LSTM2*, which attained $R^{2}$ values of 0.73, whereas the original *LSTM* model recorded an $R^{2}$ of 0.71. Expanding on their research, the same authors further developed and compared advanced machine learning models for ${\mathrm{PM}}_{2.5}$ prediction in Kaohsiung city (Taiwan) [38]. Several techniques were assessed, including *Gradient-Boosted Tree (GBT)*, *Support Vector Regression (SVR)*, *Long Short-Term Memory (LSTM)*, and an enhanced model called *Aggregated LSTM (ALSTM)*. Their findings revealed that *ALSTM* outperformed all other models, achieving an $R^{2}$ coefficient of 0.88. In a similar vein, Masih [39] underscored the effectiveness of advanced neural networks, particularly *ALSTM*, in refining air pollution prediction accuracy, particularly for ${\mathrm{PM}}_{2.5}$ concentrations. These results further reinforce the potential of deep learning methodologies in addressing urban air quality challenges.

Deep learning architectures, such as *LSTM (Long Short-Term Memory)* and *ConvLSTM (Convolutional LSTM)*, have been developed to enhance the predictive accuracy of air pollutants, including ${\mathrm{PM}}_{2.5}$, ${\mathrm{NO}}_{2}$, $O_{3}$, and ${\mathrm{SO}}_{2}$. A notable application of these models in Beijing is presented in the work of Mokhtari et al. [40], where data from local monitoring stations were utilized to train and evaluate various predictive approaches. Comparisons were conducted between traditional models, such as *Support Vector Regression (SVR)* and *Random Forest (RF)*, and more advanced deep learning techniques.

*Gated Recurrent Unit (GRU)*-based models have also been employed to predict ${\mathrm{PM}}_{2.5}$ concentrations across different regions, utilizing data from 67 monitoring stations in Taiwan. These GRU models were further enhanced through adaptive weighting techniques and spatio-temporal feature integration, leading to improved predictive performance. The findings of Lin et al. [41] highlight the effectiveness of *GRU* models in forecasting air pollution in urban areas.

Various air quality prediction models have been developed using advanced artificial intelligence techniques, including *Long Short-Term Memory (LSTM)* and *Particle Swarm Optimization (PSO)*, in conjunction with traditional models such as *Support Vector Machine (SVM)* and *Generalized Additive Model (GAM)*. These methodologies have been applied to estimate pollutant concentrations, including PM2.5, PM10, ${\mathrm{NO}}_{2}$, CO, $O_{3}$, and ${\mathrm{SO}}_{2}$ [42]. The results indicate that the *LSTM-PSO* model achieved superior accuracy compared to other models, such as *Gradient-Boosted Decision Trees (GBDT)*.

For short-term forecasts ranging from 1 to 24 h, ${\mathrm{PM}}_{2.5}$ concentration prediction models have also been developed using deep neural networks, including *Convolutional Neural Networks (CNNs)*, *LSTM*, and *CNN-LSTM* [43]. The findings demonstrate that deep learning models significantly outperform traditional approaches in predictive accuracy, with *LSTM* and *CNN-LSTM* models achieving the highest performance, yielding $R^{2}$ values exceeding 0.92. An insightful study was conducted in Ahvaz (Iran), a city notorious for its high pollution levels due to frequent dust storms [44]. This research analyzed a full year of data to evaluate the effectiveness of an *Artificial Neural Network (ANN)* model in estimating pollutant concentrations, including $O_{3}$, ${\mathrm{NO}}_{2}$, ${\mathrm{SO}}_{2}$, PM10, PM2.5, and CO. The results demonstrated that the *ANN* model achieved a mean accuracy of $R^{2}=0.87$ across all pollutants, proving to be particularly effective in predicting the *Air Quality Health Index (AQHI)*. Consequently, the study concluded that air quality authorities could leverage *ANN*-based models to forecast spatial and temporal pollution patterns, thereby mitigating adverse public health effects.

In a related study conducted in Tehran (Iran), the predictive accuracy of *Multiple Linear Regression (MLR)* and *Artificial Neural Network (ANN)* models for daily ${\mathrm{NO}}_{2}$ concentrations was assessed [45]. This analysis incorporated meteorological, urban traffic, and green space data collected over a one-year period. The findings indicated that the *ANN* model significantly outperformed the *MLR* model in predictive accuracy. Furthermore, the study highlighted the crucial role of urban green spaces in reducing ${\mathrm{NO}}_{2}$ concentrations, demonstrating their effectiveness as a more impactful factor than traffic volume. Another significant study conducted in Los Angeles (USA) compared the performance of *Land-Use Regression (LUR)* and a *2-step approach (UK2)* in predicting summertime ${\mathrm{NO}}_{x}$ concentrations [46]. This research represented one of the pioneering applications of land-use regression models for traffic-related ${\mathrm{NO}}_{x}$, ${\mathrm{NO}}_{2}$, and ${\mathrm{NO}}_{x}$ air pollution in Los Angeles. The study achieved a prediction accuracy of 87–91% by incorporating remotely sensed variables as effective surrogates, ultimately surpassing the accuracy of traditional land use-based regression models [47].

## 3. Methods

Building on the previous comparative analysis, this chapter addresses RQ2 by applying predictive models to quantify and anticipate the reduction in air pollution resulting from the implementation of the bicycle-sharing system in Madrid. This approach provides a data-driven framework for assessing the environmental benefits of sustainable urban mobility solutions, offering valuable insights for policymakers and urban planners. The city of Madrid is the most populous urban center in Spain, with approximately 3.4 million inhabitants, while the greater Madrid region has a population of around 7.0 million. This accounts for approximately 7.0% and 14.2% of the total Spanish population, respectively. Given this demographic concentration, Madrid is a significant contributor to national ${\mathrm{CO}}_{2}$ emissions from transportation. Since cycling presents a viable and sustainable alternative to conventional road transport, it becomes essential to estimate the reduction in greenhouse gas emissions facilitated by the adoption of bicycle-sharing systems.

The model described below follows a structured process. First, the raw travel data are preprocessed by filtering out extreme durations and estimating distances. They are then aggregated on a weekly basis. Next, a univariate time series is constructed and the seasonal pattern is estimated. Then, the ARNN model is configured by means of grid search and cross-validation to capture non-linear trends. Finally, after validation, the bike-sharing usage is forecasted, translating the results into estimated CO_2_ and NO_x_ emission savings.

### 3.1. Data

The dataset utilized in this study was sourced from the open data portal of the transport company of Madrid [48]. This repository provides regularly updated data with the latest available records. To facilitate a comprehensive temporal analysis, a full-year dataset was selected, making 2022 the most recent complete year for evaluation. The dataset comprised a total of 3,277,177 observations across 17 variables (cf. Table A2).

For the initial data exploration, analysis, and predictive modeling, RStudio (version 4.2.3) was chosen due to its open-source nature and specialization in handling large-scale data analysis. Additionally, RMarkdown was employed for integrating textual documentation with R code, allowing for seamless reproducibility. This interface is based on the Markdown language, designed to efficiently convert plain text into HTML format with minimal complexity. To analyze and visualize the bike-sharing system network structure, the Gephi (version 0.10) API was utilized. This open-source, Java-based interactive tool is well suited for managing large datasets and analyzing complex systems characterized by dynamic and hierarchical graphs [49]. Gephi was selected for its ability to enable real-time interaction, allowing users to modify both the properties and visual representations of the studied graphs. This feature is particularly valuable for optimizing the routing and operational efficiency of the bike-sharing service [50].

The study population included all users who utilized the bike-sharing service during the selected period. To ensure methodological consistency, it was estimated that urban bicycle trips typically last between 2 and 30 min. Trips with durations shorter than 2 min were presumed to be due to user errors in undocking the bicycle as reaching another station within such a short time was highly improbable. Conversely, trips exceeding 30 min were excluded to ensure that the entire journey was conducted solely by bicycle, without prolonged stops, aligning with the characteristics of urban mobility patterns.

Before the models were trained, the dataset was preprocessed. This included (i) the exclusion of anomalous records, specifically trips of less than 2 min (often caused by decoupling errors) and more than 30 min (to ensure that complete trips were made only by bicycle); (ii) the transformation of time stamps into categorical variables such as time of day and day of the week; and (iii) the estimation of distance traveled based on trip duration and an average bicycle speed of 12 km/h, as recommended by [51].

### 3.2. Materials

Artificial neural networks (ANNs) are fundamental algorithms for analyzing the relationship between logistics performance, energy consumption, and environmental degradation [52]. The model developed in this study is based on a multilayer perceptron (MLP) architecture, which is widely employed for nonlinear time series prediction [53]. This specific configuration of autoregressive neural networks (ARNNs) is derived by integrating a linear autoregressive model with an MLP [54]. The model relies on a statistical nonlinearity contrast, where both models are compared to determine their effectiveness [55,56,57]. In an ARNN framework, the dependent variable ($y_{t}$) is obtained as a nonlinear function of its *P* past values: $y_{t-p}$, for p=1,…,P, as expressed in the following equation:

(1) $$y_{t}^{*}=\eta +\sum_{p=1}^{P}\varphi_{p}y_{t-p}+\sum_{h=1}^{H}\beta_{h}G\left(\omega_{h}+\sum_{p=1}^{P}\alpha_{p,h}y_{t-p}\right)$$

where G() is the sigmoid function [58,59,60]. The parameters of the model—η, $\varphi_{p}$, $\beta_{h}$, $\omega_{h}$, and $\alpha_{p,h}$ for i=1,…,P and h=1,⋯,H—are estimated by minimizing the regularization error: $\lambda \cdot E_{*}$. It should be noted that λ is a user-predefined external parameter; $e_{t}$ represents the errors between the forecast $y_{t}^{*}$ and the desired value $y_{t}$. Also, $E_{*}$ is a function of the model parameters:

(2) $$E_{*}=\left|\eta \right|+\sum_{h=1}^{H}\left(|\beta_{h}\left|+\right|\omega_{h}|\right)+\sum_{p=1}^{P}|\varphi_{p}|+\sum_{p=1}^{P}\sum_{h=1}^{H}\left|\alpha_{p,h}\right|$$

The model described in Equation (1) can be reduced to a standard multilayer perception if the constraint $\varphi_{1}=\varphi_{2}=\cdots =\varphi_{P}$ is imposed. Conversely, the neural network simplifies to an autoregressive model when the condition H=0 is enforced. Finally, during the model estimation process, several statistical measures are calculated, including variance ($\sigma^{2}$), the logarithm of the likelihood function of errors, and the values of the Akaike and Schwartz information criteria. The model implementation is carried out using the BFGS optimization algorithm, which is executed multiple times with automatic restarts to ensure convergence to the optimal model.

The ARNN model combines the strengths of classical autoregressive models with nonlinear modeling capabilities of feedforward neural networks [53,58]. Unlike traditional models such as ARIMA, which assume linearity and stationarity, ARNN models can accommodate nonlinear dynamics and seasonal trends present in urban mobility patterns.

Hyperparameter tuning was conducted using a grid search procedure with 10-fold cross-validation. The key parameters optimized were the number of input lags (*p*), the number of neurons in the hidden layer (*H*), and the seasonal period (*s*). The seasonal component was set to 7, reflecting weekly usage patterns. The optimal configuration was determined by minimizing the mean absolute percentage error (MAPE) and maximizing R^2^ using 10-fold cross-validation on the training set. The results shown in Section 4 align with prior research that demonstrates the superiority of ARNNs and other ANN-based models for environmental and mobility time series forecasting [36,53,58].

### 3.3. Procedure

This chapter describes the method used to estimate the approximate amount of energy saved in fuel consumption due to the implementation of the new bike-sharing service. Additionally, the equivalent kilograms of ${\mathrm{CO}}_{2}$ and ${\mathrm{NO}}_{x}$ emissions avoided as a result of this service are quantified. The analysis was performed following the procedure by Chen et al. [20] in New York. Although the New York system recorded a significantly higher number of trips, approximately 8.1 million compared to 3.4 million in Madrid, the methodological approach remains valid. An effective approach to assessing the environmental benefits of a bike-sharing system is by estimating the fuel savings in gasoline and diesel consumption. Based on the work of Scheiner [51], the primary modes of transport were classified according to the distance traveled (cf. Table 1). The environmental impact of walking and using shared electric bicycles was considered negligible as these modes do not contribute to fuel consumption.

A series of parameters were considered that affected the calculation of the energy consumed, such as the average fuel consumption of a bus or car, the density of the fuel, and coefficients specific to each means of transport. In addition, the value of the kilograms of ${\mathrm{CO}}_{2}$ and ${\mathrm{NO}}_{x}$ generated for each kilogram of fuel consumed was known. The value of each of the parameters can be seen in Table 2.

From these two tables, Equation (3) was obtained, which calculated the energy consumed by each vehicle:

(3) $$N=\left\{\begin{array}{c} \frac{d\cdot p_{1}\cdot \rho_{1}}{\lambda_{e_{1}}\cdot \lambda_{t_{1}}}\\ \frac{d\cdot p_{2}\cdot \rho_{2}}{\lambda_{e_{2}}\cdot \lambda_{t_{2}}} \end{array}\right.$$

where *N* is the energy consumed by the vehicle, *d* refers to the total distance traveled in km, and $p_{1}$ is the amount of diesel in liters consumed by a bus per kilometer traveled, expressed in liters per kilometer (L/km). $\rho_{1}$ represents the density of diesel, measured in kilograms per liter (kg/L). These values differ between buses (diesel-powered) and private cars (gasoline-powered). Both $\lambda_{e_{1}}$ and $\lambda_{t_{1}}$ are dimensionless and express the energy efficiency produced by the fuel in the explosion in the engine and in the direct transmission to the vehicle’s transport. The second part of this expression ($p_{2}$, $\rho_{2}$, $\lambda_{e_{2}}$, and $\lambda_{t_{2}}$) represents the same for gasoline combustion in cars. This study focused particularly on the reduction of ${\mathrm{CO}}_{2}$ and ${\mathrm{NO}}_{x}$ emissions. The kilograms saved of these pollutant gases could be calculated using Equation (4):

(4) $$E=\left\{\begin{array}{c} d\cdot p_{1}\cdot \rho_{1}\cdot f_{i}\\ d\cdot p_{2}\cdot \rho_{2}\cdot f_{i} \end{array}\right.$$

where *E* represents the amount of emissions in kilograms of ${\mathrm{CO}}_{2}$ and ${\mathrm{NO}}_{x}$ that is saved from being discharged into the atmosphere. *d*, *p*, and ρ are the same parameters of the previous formula and $f_{i}$ refers to the contribution factor corresponding to each of the polluting gases, i.e., the amount of ${\mathrm{CO}}_{2}$ or ${\mathrm{NO}}_{x}$ emitted per kilogram of fuel consumed.

### 3.4. Analysis

The distance between stations along each route was calculated using the longitude and latitude coordinates of each station. Since this method does not account for actual urban routes, it provided only an approximate estimation. A more accurate measure was obtained by considering the duration of each trip (available with precise data) and the average speed of the bicycles. An average speed of 12 km/h was assumed, a value supported by various reports and media sources from the Madrid Transport Consortium [61]. Based on this speed and the recorded travel time, a reliable estimate of the actual distance traveled for each route was obtained. To estimate the equivalent trips that would have been made by car or bus, Table 1 was used, which provided the percentage of trips that in the absence of the bike-sharing service, would have relied on motorized vehicles. For example, if a trip lasted 13.5 min, the estimated distance was 2.7 km (assuming an average speed of 12 km/h). This distance falls within the 2–3 km range in Table 1, which corresponds to 7% of trips that would have been taken by bus and 68% by car.

**Figure A1:**
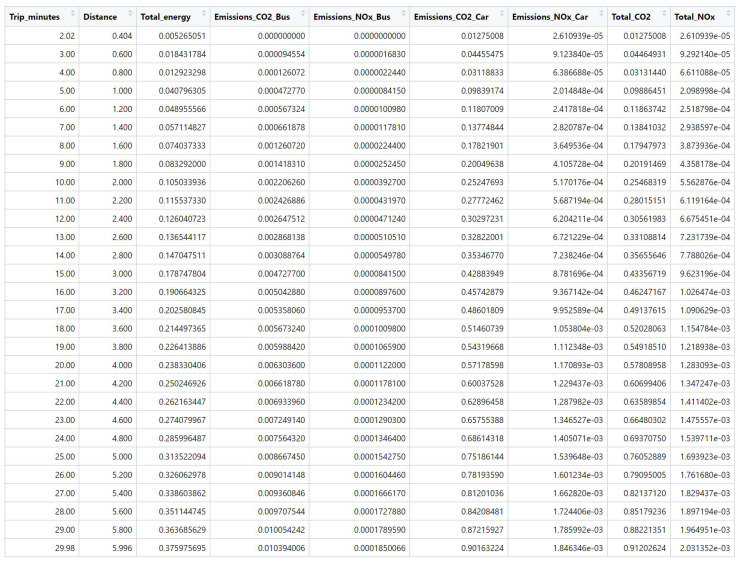
Sample of equivalent energy savings of ${\mathrm{CO}}_{2}$ and ${\mathrm{NO}}_{x}$.

Using these proportions, it was estimated that the avoided emissions corresponded to a 189-meter bus trip (7% of 2.7 km) and a 1.84 km car trip (68% of 2.7 km). The remaining 25% of trips were considered non-polluting as they would have been completed by bicycle or on foot. By applying these parameters and the proportion of distances traveled by different polluting vehicles, the values were incorporated into the pollution estimation equation. This method enabled the calculation of the equivalent amount of energy saved, as well as the reduction in ${\mathrm{CO}}_{2}$ and ${\mathrm{NO}}_{x}$ emissions for each trip. A sample of observations detailing the total distance of each trip and the corresponding emissions savings is presented in Figure A1 Appendix B.

## 4. Results

Understanding the travel patterns and usage profiles of a bike-sharing system is essential for optimizing its operation and expanding its impact on urban mobility. This chapter analyzes various trip characteristics, such as duration, distance, and frequency, as well as the times of day and days of the week when the BSS was most frequently used. By examining these factors, we aim to identify key patterns that inform user behavior and reveal opportunities for improving service efficiency.

**Figure 2:**
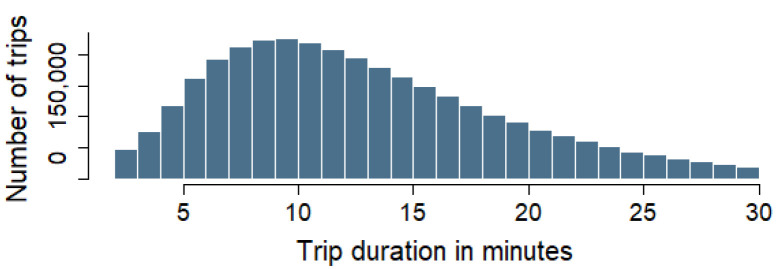
Histogram of trip duration.

The histogram in Figure 2 shows the distribution of BSS trip durations in Madrid. The shape of the histogram reveals that most trips lasted between 10 and 15 min, with a peak in that range. After 15 min, the frequency of trips gradually decreased, with very few trips exceeding 30 min. This suggests that most users take relatively short trips. A closer analysis indicates that 50% of trips lasted between approximately 7 and 15 min, with a median of around 10 min. Additionally, the majority of trips fell within the range of 5 to 25 min. Trips lasting around 30 min were considered unusually long and treated as outliers. These results may have been influenced by the fact that the BSS in Madrid offers a flat rate of EUR 10 per month, provided that trips last less than 30 min. Any trip exceeding this duration incurs additional charges for the extra time. An exhaustive analysis of the BSS in Madrid is given in Appendix D.

### 4.1. Quantifying Greenhouse Gas Emission Savings

**Figure 3:**
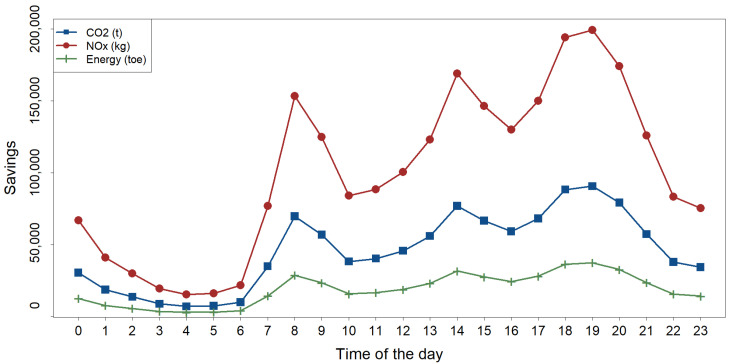
Equivalent energy savings for ${\mathrm{CO}}_{2}$ and ${\mathrm{NO}}_{x}$ distributed by time of day.

The distribution of energy and pollutant gas savings throughout the day was examined. Figure 3 illustrates how these savings were concentrated during peak bicycle usage hours, which coincided with the peak demand for other motorized transport modes. The results highlight that the BSS not only contributed to reducing GHG emissions but also prevented additional emissions from being generated during periods of high traffic and pollution. This method quantified savings at individual and grouped docking station levels, offering valuable insights for urban mobility planning.

The use of electric bicycles in Madrid’s bike-sharing system resulted in savings of approximately 451.8 tons of oil equivalent, corresponding to 1095.7 tons of CO_2_ and approximately 2.4 tons of NO_*x*_ in 2023. Our method enabled us to estimate the reduction in greenhouse gas emissions for groups of docking stations. For instance, an analysis of the stations located near university schools and faculties in Ciudad Universitaria quarter revealed a total reduction of 5440 kg of emissions.

### 4.2. Anticipating Greenhouse Gas Emission Savings

**Figure 4:**
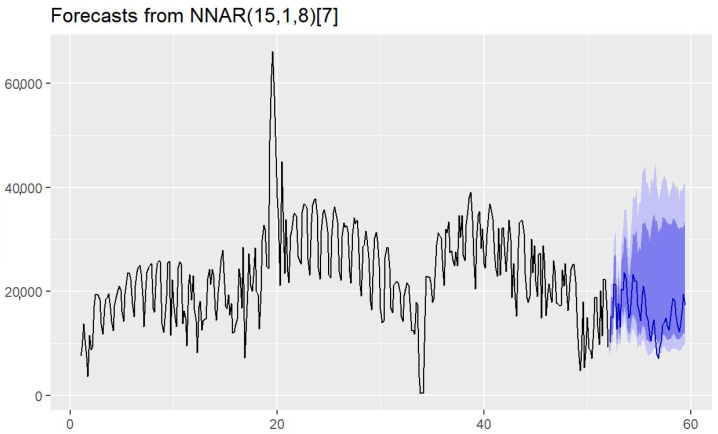
Y-axis: predicted values; X-axis: time in weeks. NNAR(15,1,8)[7] model prediction.

The review in Section 2 shows that the ARNN model is well suited for predicting GHG emissions in bike-sharing systems due to its ability to handle non-linear time series, capture seasonal patterns, and adapt to dynamic data. Its flexibility in configuring input delays and hidden neurons allows for accurate short- and long-term forecasts. Additionally, it effectively predicts peaks in bicycle usage, which coincide with reduced motorized vehicle emissions, thereby providing a reliable tool for quantifying environmental benefits from BSS. Figure 4 shows that the resulting model was an NNAR(15,1,8)[7], meaning that the model had 15 input lags, one output unit, and eight neurons in the hidden layer. The series pattern had weekly stationarity (seven periods). Examining the forecasts for the next seven weeks, it was evident that the model performed well. In general, ARNN models effectively capture the asymmetry of cycles better than classical models such as ARIMA. The use of ARNN models in this study enabled the capture of nonlinear patterns and seasonal trends in emission reductions attributable to the BSS. Compared to traditional regression-based approaches, our model enhances prediction accuracy and provides a scalable methodology for other cities with available IoT data.

The prediction obtained with the model proved to be highly accurate. The large amount of data available for training significantly improved the results of the analysis. In this case, the model had a low mean absolute percentage error (MAPE = 7%), suggesting that it performed quite well in percentage terms. The mean scaled absolute error (MASE) below 1 indicates that the model is better than a naive model. Finally, the fit of the model to the data was good ($R^{2}=0.94$) and the value of the residual variance showed that the errors were small ($\sigma^{2}=0.02242$).

The predictive model enabled the calculation of the total distance expected to be covered in the forecasted weeks. According to this model, based on 52 weeks, the total estimated distance was nearly 200 km. From this data, further estimates of energy savings and greenhouse gas emissions could be calculated. Hence, it was estimated that in the first two weeks of 2023, approximately 10.8 tons of oil equivalent, 58 kg of ${\mathrm{NO}}_{x}$, and 26.2 tons of ${\mathrm{CO}}_{2}$ were saved.

## 5. Discussion and Conclusions

This work investigated the state of the art of predictive models for GHG emissions in large cities, with a focus on bike-sharing systems. The literature review highlighted four key benefits of bike-sharing systems: (i) a reduction in air pollution through decreased emissions of carbon-based pollutants (CO and CO_2_), nitrogen oxides (NO and NO_2_), sulfur dioxide (SO_2_), and particulate matter (PM_10_ and PM_2.5_), which are primarily generated by fossil fuel combustion; (ii) a decrease in urban traffic congestion and associated energy consumption costs (e.g., fuel savings); (iii) improved urban space organization; and (iv) a decline in accidents, reducing both human injuries and material damages. Despite the increasing availability of mobility data and advances in machine learning, limited research has focused on predicting the impact of BSSs on greenhouse gas (GHG) emissions using machine learning techniques [19]. However, machine learning has been extensively applied to predict urban GHG emissions without explicitly considering BSSs.

This study builds upon previous research by (i) identifying key patterns in BSS usage, (ii) quantifying energy savings and reductions in GHG emissions, (iii) forecasting energy and emission savings, and (iv) presenting a scalable modeling framework applicable to various cities and emission types. A significant contribution of this research is the classification of the scientific literature into four dimensions (RQ1): (1) predictive models utilized (n=60), (2) reported accuracy levels, (3) analyzed emissions, and (4) citation frequency per study (Figure 1 and Table A1).

The findings indicate that the most frequently used predictive models include ANN, ANFIS, LSTM-PSO, MLP, and random forest models. These models are widely adopted due to their ability to handle the complexity and non-linearity of urban data, effectively capturing intricate relationships between traffic patterns, meteorological conditions, and energy consumption, key factors in GHG emissions estimation. In particular, models such as LSTM, designed for time-series forecasting, excel in both short- and long-term predictions, which is essential in dynamic urban environments where emissions fluctuate over time. Moreover, MLP and random forest are particularly well suited for processing large-scale datasets generated by urban sensors and monitoring systems without excessive data simplification. ANFIS facilitates the integration of heterogeneous data sources, improving model robustness, while LSTM-PSO enhances predictive accuracy through optimization techniques. The widespread use of these models underscores their adaptability and effectiveness in urban GHG forecasting.

The selection of the autoregressive neural network (ARNN) model in this study was not based on its novelty in algorithmic development but on its strategic suitability for this study. ARNN models are particularly effective at capturing complex temporal dependencies and nonlinear patterns in univariate time series data. This makes them well suited to modeling the dynamic behavior of bike-sharing demand and its relationship to greenhouse gas emission reduction. While ARNN architecture is not new in the machine learning literature [53,58], its application in the context of environmental forecasting for urban bike-sharing systems remains limited. Existing studies have largely focused on retrospective analyses using regression models or traditional ANNs. In contrast, this study adopted the ARNN framework to enable short-term forecasting of CO_2_ and NO_*x*_ emission reductions, incorporating both trend and seasonality in mobility data. This application demonstrates the model’s flexibility in real-world environmental assessment scenarios and offers a replicable approach for similar urban contexts.

Regarding the accuracy (R^2^ and RMSE) of predictive models, it is noteworthy that some studies with lower accuracy levels have a high number of citations. This discrepancy may indicate a research gap and presents an opportunity for further investigation into model refinement and validation.

In terms of GHG emissions, the most frequently studied pollutants are, in descending order, PM_2.5_, NO_*x*_, NO_2_, and O_3_, whereas pollutants such as NH_3_, CO_2_, and PM_10_ receive less attention. The prioritization of PM_2.5_, NOx, NO_2_, and O_3_ in GHG research is likely influenced by the relative ease and cost-effectiveness of monitoring these pollutants compared to CO_2_ and NH_3_, which require more sophisticated measurement techniques. Advanced sensor technologies readily capture PM_2.5_ and NOx, making them highly accessible for urban monitoring, especially given their well-documented impacts on public health and the environment. In contrast, CO_2_ is a global pollutant with diffuse sources, while NH_3_ emissions are more closely associated with agricultural rather than urban activities. Although PM_10_ is monitored, its health impact is generally considered less severe than PM_2.5_, which may contribute to its lower research frequency.

Using the ARNN model, this study accurately predicted greenhouse gas emission reductions attributed to the BSS (RQ2). The predictions demonstrated high accuracy using only data from the 12 weeks preceding the forecast period. Compared to other classical models, the ARIMA model produced values of R^2^ = 0.78 and MAPE = 13.4%, significantly lower than the R^2^ = 0.94 and MAPE = 7% of the ARNN model. Similarly, linear regression models performed even worse due to their inability to model temporal dependencies (R^2^ = 0.61; MAPE = 18.2%). These results are consistent with those of [19,27], who found that deep learning and neural autoregressive models outperform traditional statistical approaches in urban mobility and pollution forecasting.

The proposed predictive model is adaptable to different contexts while accounting for specific limitations in each case. For instance, it can be applied to aggregated areas (e.g., commercial centers, universities, residential zones, or business districts) and trained with varied time spans (e.g., the last 12 weeks, one year, or five years) to generate tailored predictions (e.g., for the next day, week, or two weeks). Additionally, the model can be extended to analyze alternative variables beyond NO_x_ and CO_2_ emissions. This study introduced a significant innovation in predicting the environmental impact of BSSs by integrating real-time IoT sensor data with advanced machine learning models. While previous research has estimated emission reductions retrospectively, this methodology enables forecasting trends and improving sustainable urban planning. Moreover, the combination of prediction and real-time sensors offers a replicable tool for policymakers in other cities.

Future research could leverage this approach to predict and quantify reductions in other GHG emissions (e.g., CH_4_, O_3_, CO, PM) or additional pollutants such as noise pollution. The models explored in this study provide a robust framework for quantifying the impact of sustainable mobility policies implemented by regional governments. This analysis can aid in identifying streets requiring infrastructure improvements, such as the installation of additional bike lanes, docking stations, or bicycles, and aligns closely with machine learning-based models used to assess transportation policies aimed at reducing GHG emissions.

**Figure A2:**
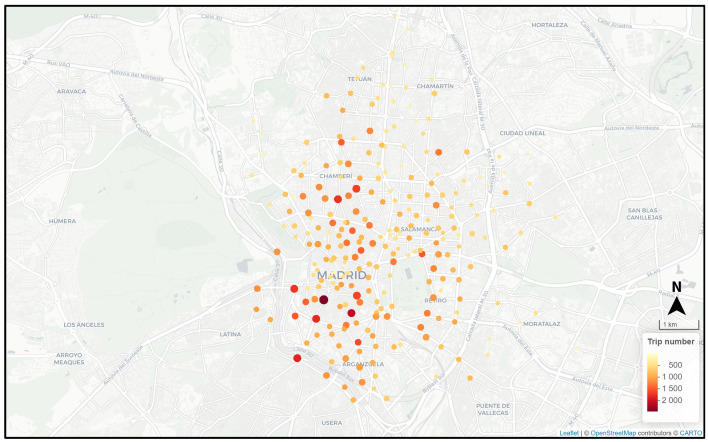
Madrid BSS station distribution (monthly data).

**Figure A3:**
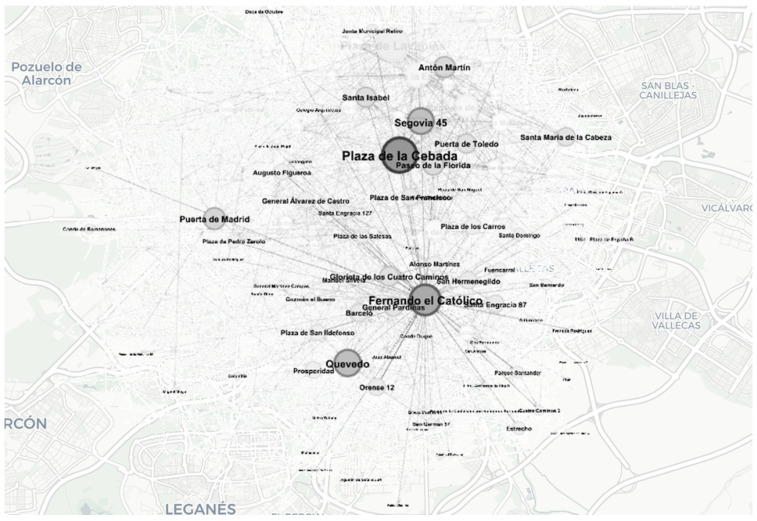
Hubs in BSS Madrid: best-connected docking stations.

The findings of this study highlight several key areas where machine learning and AI contribute significantly. Heat maps displaying the most active stations (Figure A2 and Figure A3) enable the identification and prediction of stations that may require additional bicycles at specific times of the day. This information facilitates the optimal redistribution of bicycles across stations, helping maintenance personnel minimize periods when stations either run out of bicycles or lack available docking spaces. Moreover, route optimization for maintenance staff could reduce travel time and operational costs. Time-series data on bicycle usage throughout the day also assist in determining optimal time frames for infrastructure maintenance.

Finally, this study acknowledges a limitation in the dataset as BSS data lack GPS traceability throughout each bicycle’s journey, capturing only pickup and drop-off locations. This restriction limits the ability to cross-reference data with additional sources (e.g., real-time traffic conditions, air or noise pollution levels, green routes, tourist areas, or accident-prone locations), constraining the full potential of machine learning and IoT applications in this context. In addition, possible sources of prediction bias must be taken into account. Although the model was trained with data from an entire year and performed well, certain patterns (such as night-time use or certain user groups) may have been captured with less precision. Furthermore, the use of average values for vehicle type, fuel efficiency, and emission factors introduced generalizations that may not have fully reflected real-world variability. Future research could integrate geolocated mobile and ubiquitous sensing from users’ smartphones [62], incorporating additional parameters such as noise levels, light exposure, and GPS tracking. Furthermore, enabling users to annotate routes based on their experiences (e.g., labeling routes as safe/unsafe, conducive to physical activity, cyclist-adapted, or tourist-friendly) [63] could provide valuable insights into urban mobility preferences and enhance the functionality of bike-sharing systems.

## Figures and Tables

**Table 1:** Distribution of the percentage of transportation mode usage based on distance traveled.

km	On Foot	Bicycle	Bus	Car
≤0.2	94%	5%	0%	1%
0.2–0.4	81%	11%	0%	7%
0.4–0.6	64%	19%	0%	17%
0.6–0.8	38%	19%	1%	40%
0.8–1	56%	21%	1%	21%
1.0–1.5	25%	19%	3%	53%
1.5–2.0	18%	17%	5%	60%
2–3	10%	14%	7%	68%
3–5	4%	9%	10%	77%
5–7	1%	6%	11%	81%
7–10	1%	4%	12%	82%
10–20	0%	2%	10%	87%
>20	1%	1%	13%	85%

**Table 2:** Units of consumption and pollution for motorized vehicles: bus and car parameters. Source: Chen et al. [20].

Symbol	Parameters	Units	Bus	Car
p	Fuel consumption	L/km	0.006	0.088
ρ	Fuel density	kg/L	0.85	0.72
$\lambda_{e}$	Combustion efficiency	—	0.93	0.87
$\lambda_{t}$	Transport efficiency	—	0.99	0.95
${\mathrm{CO}}_{2}$	Carbon dioxide emission factor	kg/kg	3.09	2.93
${\mathrm{NO}}_{x}$	Emission factor for nitrogen oxides	kg/kg	0.055	0.006

**Figure A4:**
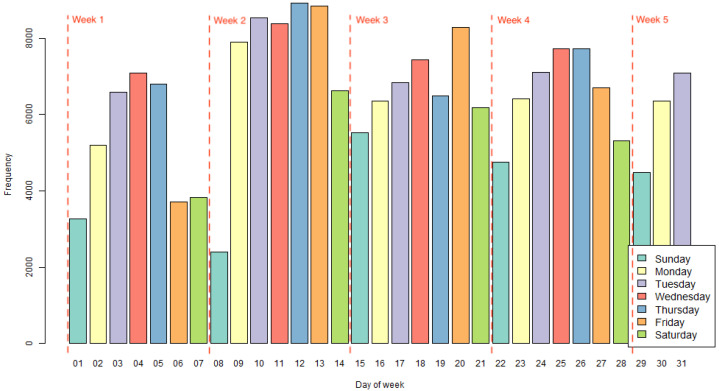
Number of trips on each day of the month colored according to the day of the week.

**Figure A5:**
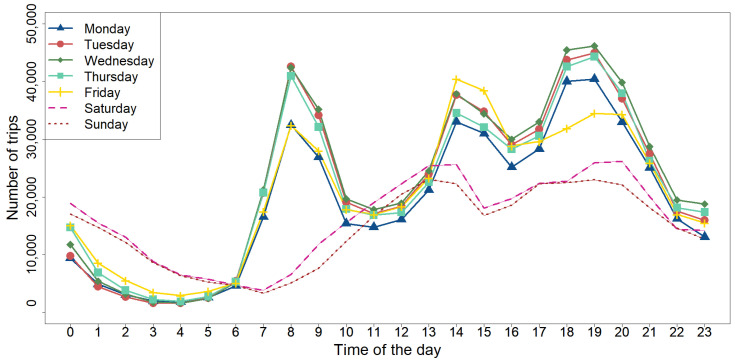
Frequency divided by hours per day.

**Figure A6:**
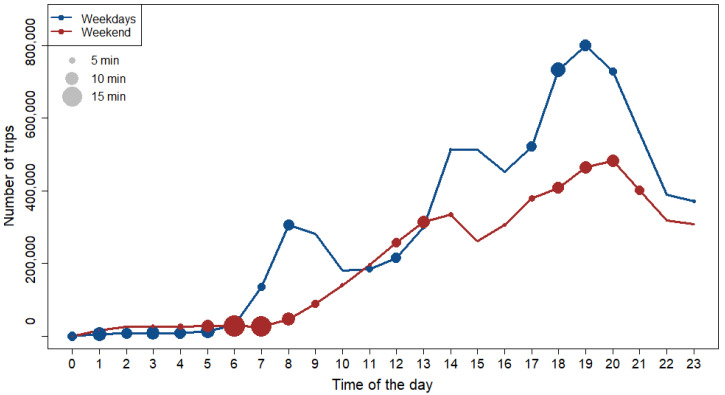
Average weekday and weekend frequency divided by hours per day and duration.

## Data Availability

The data presented in this study are available on request from the corresponding author.

## Appendix A. Machine Learning Models Anticipating Pollutants in Urban Areas

**Table A1:** Review of ML models that evaluate urban pollutants and their accuracy.

Reference	Model	Pollutants	Accuracy $R^{2}$/RMSE
Zhang et al. [24]	TROPOMI	${\mathrm{CO}}_{2}$	0.97 / —
	TROPOMI	${\mathrm{NO}}_{x}$	0.97 / —
Li et al. [26]	SVM	PM2.5	0.75 / —
	GAM	${\mathrm{PM}}_{2.5}$	0.76 / —
	RF	${\mathrm{PM}}_{2.5}$	0.83 / —
	BRT	${\mathrm{PM}}_{2.5}$	0.83 / —
	XGBoost	${\mathrm{PM}}_{2.5}$	0.82 / —
	Cubist	${\mathrm{PM}}_{2.5}$	0.81 / —
	SVM	${\mathrm{NO}}_{x}$	0.69 / —
	GAM	${\mathrm{NO}}_{x}$	0.59 / —
	RF	${\mathrm{NO}}_{x}$	0.71 / —
	BRT	${\mathrm{NO}}_{x}$	0.71 / —
	XGBoost	${\mathrm{NO}}_{x}$	0.70 / —
	Cubist	${\mathrm{NO}}_{x}$	0.70 / —
Wen et al. [27]	BRT	${\mathrm{NO}}_{x}$	0.93 / —
	BRT	${\mathrm{CO}}_{2}$	0.9 / —
Zeinalnezhad et al. [28]	ANFIS	CO	0.8693 / —
	RS	CO	0.8445 / —
	ANFIS	${\mathrm{SO}}_{2}$	0.8011 / —
	RS	${\mathrm{SO}}_{2}$	0.8001 / —
	ANFIS	O_3_	0.8350 / —
	RS	O_3_	0.7830 / —
	ANFIS	NO_2_	0.7640 / —
	RS	NO_2_	0.7602 / —
Arhami et al. [29]	ANN	CO	0.92 / —
	ANN	O_3_	0.77 / —
	ANN	${\mathrm{PM}}_{10}$	0.87 / —
	ANN	${\mathrm{NO}}_{x}$	0.87 / —
	ANN	${\mathrm{NO}}_{2}$	0.85 / —
	ANN	NO	0.82 / —
Goulier et al. [30]	MLP	${\mathrm{CO}}_{2}$	0.685 / —
	MLP	${\mathrm{NH}}_{3}$	0.648 / —
	MLP	NO	0.751 / —
	MLP	${\mathrm{NO}}_{2}$	0.915 / —
	MLP	${\mathrm{NO}}_{x}$	0.751 / —
	MLP	O_3_	0.871 / —
	MLP	${\mathrm{PM}}_{10}$	0.315 / —
	MLP	${\mathrm{PM}}_{2.5}$	0.587 / —
	MLP	${\mathrm{PM}}_{10}$	0.536 / —
	MLP	${\mathrm{PM}}_{10}$	0.449 / —
Shi and Harrison [31]	OLS	${\mathrm{NO}}_{2}$	0.83 / —
	AR	${\mathrm{NO}}_{x}$	0.65 / —
Vasseur and Aznarte [32]	QRF	${\mathrm{NO}}_{2}$	— / 22.62
	QRFL	${\mathrm{NO}}_{2}$	— / 19.12
	QKNN	${\mathrm{NO}}_{2}$	— / 20.73
	QKNNL	${\mathrm{NO}}_{2}$	— / 18.4
	QGB	${\mathrm{NO}}_{2}$	— / 16.09
	QGBL	${\mathrm{NO}}_{2}$	— / 16.14
	QLR	${\mathrm{NO}}_{2}$	— / 19.44
	MLP	${\mathrm{NO}}_{2}$	— / 18.16
	NGBOOST	${\mathrm{NO}}_{2}$	— / 18.52
	DT	${\mathrm{NO}}_{2}$	— / 18.69
Liu et al. [33]	EWT-MAEGA-NARX	${\mathrm{PM}}_{2.5}$	— / 0.1793
	EWT-MAEGA-NARX	${\mathrm{SO}}_{2}$	— / 0.0347
	EWT-MAEGA-NARX	${\mathrm{NO}}_{2}$	— / 0.0969
	EWT-MAEGA-NARX	CO	— / 0.0041
	VMD-MAEGA-NARX	${\mathrm{PM}}_{2.5}$	— / 0.3361
	EWT-MAEGA-SVM	${\mathrm{NO}}_{2}$	— / 0.9451
	EWT-ARIMA-NARX	${\mathrm{NO}}_{2}$	— / 0.3548
Mercer et al. [47]	UK	${\mathrm{NO}}_{x}$	0.75 / —
	LUR	${\mathrm{NO}}_{x}$	0.74 / —
	UK2	${\mathrm{NO}}_{x}$	0.75 / —
Su et al. [46]	LUR (ADDRESS)	NO	0.81 / —
	LUR (ADDRESS)	${\mathrm{NO}}_{2}$	0.86 / —
	LUR (ADDRESS)	${\mathrm{NO}}_{x}$	0.85 / —
Wen et al. [36]	C-LSTME	${\mathrm{PM}}_{2.5}$	— / 12.08
	ST-C-LSTM	${\mathrm{PM}}_{2.5}$	— / 17.76
	LSTME	${\mathrm{PM}}_{2.5}$	— / 18.25
	LSTME (AOD)	${\mathrm{PM}}_{2.5}$	— / 21.17
	LSTME (Meteo)	${\mathrm{PM}}_{2.5}$	— / 22.22
	LSTM NN	${\mathrm{PM}}_{2.5}$	— / 25.95
	ARMA	${\mathrm{PM}}_{2.5}$	— / 34.40
	SVR	${\mathrm{PM}}_{2.5}$	— / 39.92
Mao et al. [34]	TS-LSTME	${\mathrm{PM}}_{2.5}$	0.72 / —
	TS-LSTME	O_3_	0.86 / —
	LSTME	${\mathrm{PM}}_{2.5}$	0.52 / —
	LSTME	O_3_	0.63 / —
	LSTM	${\mathrm{PM}}_{2.5}$	0.52 / —
	LSTM	O_3_	0.60 / —
Chang et al. [37]	GBT	${\mathrm{PM}}_{2.5}$	0.83 / —
	SVR	${\mathrm{PM}}_{2.5}$	0.73 / —
	LSTM	${\mathrm{PM}}_{2.5}$	0.71 / —
	LSTM2	${\mathrm{PM}}_{2.5}$	0.73 / —
Chang et al. [38]	GBT	${\mathrm{PM}}_{2.5}$	0.86 / —
	SVR	${\mathrm{PM}}_{2.5}$	0.87 / —
	LSTM	${\mathrm{PM}}_{2.5}$	0.85 / —
	ALSTM	${\mathrm{PM}}_{2.5}$	0.88 / —
Rybarczyk and Zalakeviciute [64]	GBT	${\mathrm{PM}}_{2.5}$	— / 1.59
	SVR	${\mathrm{PM}}_{2.5}$	— / 2.77
	LSTM	${\mathrm{PM}}_{2.5}$	— / 1.59
	ALSTM	${\mathrm{PM}}_{2.5}$	— / 0.44
Masih [39]	LSTM	${\mathrm{PM}}_{2.5}$	0.78 / —
	SVR	${\mathrm{PM}}_{2.5}$	0.73 / —
	GBTR	${\mathrm{PM}}_{2.5}$	0.76 / —
	ALSTM	${\mathrm{PM}}_{2.5}$	0.82 / —
Mokhtari et al. [40]	LSTM	${\mathrm{PM}}_{2.5}$	0.91 / —
	SVR	${\mathrm{NO}}_{2}$	0.85 / —
	ANN	O_3_	0.89 / —
	RF	${\mathrm{SO}}_{2}$	0.82 / —
	GBDT	CO	0.87 / —
	CNN	${\mathrm{NO}}_{x}$	0.88 / —
Lin et al. [41]	GRU-13d	${\mathrm{PM}}_{2.5}$	0.91 / —
	GRU-AW14d	${\mathrm{PM}}_{2.5}$	0.85 / —
	GRU-ST13d	${\mathrm{PM}}_{2.5}$	0.78 / —
	MLEGRU	${\mathrm{PM}}_{2.5}$	0.88 / —
Al-Janabi et al. [65]	LSTM-PSO	${\mathrm{PM}}_{2.5}$	0.85 / —
	LSTM-PSO	${\mathrm{PM}}_{10}$	0.85 / —
	LSTM-PSO	${\mathrm{NO}}_{2}$	0.85 / —
	LSTM-PSO	CO	0.85 / —
	LSTM-PSO	O_3_	0.85 / —
	SVM	${\mathrm{PM}}_{2.5}$	0.78 / —
	GAM	${\mathrm{NO}}_{2}$	0.81 / —
	GBDT	O_3_	0.82 / —
Mishra and Goyal [66]	PCA-ANN	${\mathrm{NO}}_{2}$	0.318 / —
Freeman et al. [67]	RNN-LSTM	O_3_	— / 2.5
Qin et al. [68]	CNN+LSTM	${\mathrm{PM}}_{2.5}$	— / 14.3
Sun and Sun [69]	CS-LSSVM	${\mathrm{PM}}_{2.5}$	— / 14.47
	LSSVM	${\mathrm{PM}}_{2.5}$	— / 21.75
	GRNN	${\mathrm{PM}}_{2.5}$	— / 22.89
Yang et al. [43]	CNN	${\mathrm{PM}}_{2.5}$	0.931 / —
	LSTM	${\mathrm{PM}}_{2.5}$	0.92 / —
	CNN-LSTM	${\mathrm{PM}}_{2.5}$	0.92 / —
	BPNN	${\mathrm{PM}}_{2.5}$	0.875 / —
Maleki et al. [44]	ANN	O_3_	0.90 / —
	ANN	${\mathrm{NO}}_{2}$	0.91 / —
	ANN	${\mathrm{SO}}_{2}$	0.99 / —
	ANN	${\mathrm{PM}}_{10}$	0.91 / —
	ANN	${\mathrm{PM}}_{2.5}$	0.91 / —
	ANN	CO	0.94 / —
Shams et al. [45]	ANN	${\mathrm{NO}}_{2}$	0.89 / —
	MLR	${\mathrm{NO}}_{2}$	0.81 / —
	MLP	${\mathrm{NO}}_{2}$	0.89 / —

## Appendix B. Equivalent Energy Savings

Figure A1 is a sample of observations showing the total distance traveled and the different emissions saved on each of these trips. If all the individual observations are added up, the total savings in terms of energy and pollutant emissions can be arrived at, thanks to the BSS (cf. Figure 3).

## Appendix C. Structure of the Working Dataset

**Table A2:** Summary of study variables.

CHARACTER				
**skim_variable**	**n_missing**	**complete_rate**	**min**	**max**
geolocation_unlock	0	1.0	53	75
address_unlock	0	1.0	15	68
geolocation_lock	0	1.0	53	75
address_lock	0	1.0	15	68
unlock_station_name	0	1.0	10	58
lock_station_name	0	1.0	10	58
day	0	1.0	2	2
hour	0	1.0	2	2
daysweek	0	1.0	5	9
**DATE**				
**skim_variable**	**n_missing**	**complete_rate**	**min**	**max**
date	0	1.0	2023-01-01	2023-01-31
**FACTOR**				
**skim_variable**	**n_missing**	**complete_rate**	**ordered**	**n_unique**
station_unlock	0	1.0	FALSE	260
dock_unlock	0	1.0	FALSE	30
station_lock	0	1.0	FALSE	260
dock_lock	0	1.0	FALSE	30
**NUMERIC**				
**skim_variable**	**n_missing**	**complete_rate**	**mean**	**sd**
trip_minutes	0	1.0	12.20849	5.980459
**POSIXCT**				
**skim_variable**	**n_missing**	**complete_rate**	**min**	**max**
unlock_date	0	1.0	2022-01-01 00:01:45	2022-12-31 23:59:44
lock_date	0	1.0	2022-01-01 00:10:05	2022-12-31 19:02:43

## Appendix D. Extended Analysis of BSS in Madrid

### Appendix D.2. Connectivity of Docking Stations

*Hubs*, or better-connected stations, are those that serve as the origin or destination for routes involving a larger number of distinct stations.

### Appendix D.3. Weekly and Monthly Usage Patterns

Figure A4 illustrates the distribution of trips from Sunday to Saturday (grouped by color) in terms of total trips over the course of a year. The data also reflect the evolution of trips across the 4–5 weeks within each month.

### Appendix D.4. Daily Usage Patterns

Figure A5 illustrates the total number of trips made throughout the day and their hourly distribution. Additionally, differences in usage patterns across the days of the week are explored to identify variations in the timing and frequency of trips.

### Appendix D.5. Leisure and Working Days

Figure A6 shows usage patterns throughout the day, distinguishing between weekdays (Monday to Friday) and non-working days (weekends). Additionally, it explores the dimension of trip duration, analyzing how travel times vary across different days of the week and time periods.
